# Passive Immunotherapy Protects against Enteric Invasion and Lethal Sepsis in a Murine Model of Gastrointestinal Anthrax

**DOI:** 10.3390/toxins7103960

**Published:** 2015-09-29

**Authors:** Bruce Huang, Tao Xie, David Rotstein, Hui Fang, David M. Frucht

**Affiliations:** 1 Division of Biotechnology Review and Research II, Office of Biotechnology Products, Office of Product Quality, Center for Drug Evaluation and Research, U.S. Food and Drug Administration, Silver Spring, MD 20993-0002, USA; E-Mails: bruce.huang@fda.hhs.gov (B.H.); tao.xie@fda.hhs.gov (T.X.); hui.fang@fda.hhs.gov (H.F.); 2 Division of Compliance, Center for Veterinary Medicine, U.S. Food and Drug Administration, Rockville, MD 20855, USA; E-Mail: david.rotstein@fda.hhs.gov

**Keywords:** gastrointestinal anthrax, anthrax toxin, protective antigen, monoclonal antibody, raxibacumab, lethal factor, edema factor

## Abstract

The principal portal for anthrax infection in natural animal outbreaks is the digestive tract. Enteric exposure to anthrax, which is difficult to detect or prevent in a timely manner, could be exploited as an act of terror through contamination of human or animal food. Our group has developed a novel animal model of gastrointestinal (GI) anthrax for evaluation of disease pathogenesis and experimental therapeutics, utilizing vegetative *Bacillus anthracis* (Sterne strain) administered to A/J mice (a complement-deficient strain) by oral gavage. We hypothesized that a humanized recombinant monoclonal antibody (mAb) * that neutralizes the protective antigen (PA) component of *B. anthracis* lethal toxin (LT) and edema toxin (ET) could be an effective treatment. Although the efficacy of this anti-anthrax PA mAb has been shown in animal models of inhalational anthrax, its activity in GI infection had not yet been ascertained. We hereby demonstrate that passive immunotherapy with anti-anthrax PA mAb, administered at the same time as gastrointestinal exposure to *B. anthracis*, prevents lethal sepsis in nearly all cases (>90%), while a delay of up to forty-eight hours in treatment still greatly reduces mortality following exposure (65%). Moreover, passive immunotherapy protects against enteric invasion, associated mucosal injury and subsequent dissemination by gastrointestinal *B. anthracis*, indicating that it acts to prevent the initial stages of infection. * Expired raxibacumab being cycled off the Strategic National Stockpile; biological activity confirmed by *in vitro* assay.

## 1. Introduction

Incidental cutaneous or inhalational human infections with *Bacillus anthracis* (Gram-positive spore-forming aerobic or facultative anaerobic bacteria) have intermittently occurred in agricultural regions for millennia, largely due to direct exposures associated with the husbandry, consumption or pelt preparation of domesticated ruminant herbivores [1]. The deliberate human use of anthrax has emerged as a biological terrorism threat, as well, highlighted by a series of deadly attacks in 2001 [2]. These acts of terror utilized anthrax spores delivered through the postal service in the form of a finely-powdered preparation that was inhaled by some victims, progressing to life-threatening pulmonary infections in 11 patients, five of whom succumbed [2,3].

Although justifiably intense scientific attention was subsequently directed toward investigating the mechanisms of inhalational anthrax infections [4,5,6], it is nevertheless generally regarded that the principal route of infection for *B. anthracis* in natural settings is by means of enteric entry [7]. Indeed, this avenue may be the route most favorable to its growth and propagation in grazing ruminant wildlife and livestock animals [7,8]. Moreover, it has been proposed that anthrax organisms could be deliberately introduced as a contaminant to the food supply of humans or agricultural animals as an act of terror [9], one that would be difficult to detect or prevent [10,11,12,13]. Understanding disease pathogenesis and treatment modalities in the setting of gastrointestinal anthrax is therefore a worthwhile objective.

To this end, we developed a novel animal model of gastrointestinal anthrax in which a lethal dose of vegetative *B. anthracis* (Sterne strain) organisms is administered to A/J mice (a strain deficient in complement component C5) by oral gavage. This model exploits the increased infectivity of vegetative anthrax bacteria over spores, as well as mimics the natural route of infection during consumption of infected meat [14]. Within days of exposure, animals in this model system acquire acute gastrointestinal pathology characterized by frank intestinal hemorrhage, edema and rapid progression to morbid septicemia [14]. In addition, hematologic dissemination of bacteria is observed in many organs, including the liver, kidney, lungs and spleen [14]. This model has been extensively characterized, and its robust and reproducible features render it a useful tool for *in vivo* efficacy investigations of experimental therapeutic modalities against gastrointestinal anthrax.

Several strategies for post-exposure treatment against *B. anthracis* infection exist. First-line therapeutics, such as the antibiotics ciprofloxacin and doxycycline, have shown effectiveness if administered in advance of symptomatic disease [15,16]. However, while critical for their bactericidal activity in clearing live *B. anthracis* organisms, antibiotic therapies are not effective in blocking the activity of the anthrax toxins that have been secreted prior to bacterial death. These toxins are responsible for many of the systemic sequelae associated with anthrax pathophysiology [17,18,19,20,21,22,23,24,25,26]. For the effective inhibition of toxin activity, a different approach is necessary, one that is specific and highly effective at blocking the assembly or cellular entry of the toxins. For this reason, immunologic modalities are of great interest to researchers, due to their inherent precision in targeting epitopes unique to anthrax toxin elements that are critical for toxin function [27].

The protective antigen (PA) component of anthrax toxin represents an attractive therapeutic target. PA performs a crucial function as a central participant in the binding and formation of lethal toxin (LT) and edema toxin (ET) at specific receptors on the surface of target cells, leading to their eventual clathrin-mediated endocytosis into the interior of the cell [27,28,29,30]. Raxibacumab, a monoclonal antibody licensed in the U.S. in 2012 for the prophylaxis and treatment of inhalational anthrax in humans, is one example of a biopharmaceutical developed to target PA [31,32]. This humanized recombinant IgG1λ monoclonal antibody has been shown to specifically recognize domain IV of the PA protein with high affinity; domain IV is critically responsible for the binding of PA to cell surface receptors [33]. Raxibacumab binding thus interferes in the interaction of PA with its receptors on the surface of target cells, thereby preventing the entry of LT and ET into the cell interior [34,35]. Raxibacumab has shown some beneficial effects in the treatment of inhalational anthrax in several animal models, including rabbits [34,36,37] and monkeys [34]. However, the value of this or other monoclonal antibodies as therapeutic agents against gastrointestinal anthrax infection had not previously been ascertained. We herein demonstrate the efficacy of passive immunotherapy with a monoclonal antibody (expired raxibacumab being cycled off the Strategic National Stockpile, but confirmed to retain biological activity (data not shown)) using our murine gastrointestinal anthrax infection model. We herein show that mice administered a single intravenous dose of this toxin-neutralizing monoclonal antibody demonstrate greatly improved survival outcomes when the drug is administered within 48 h following exposure to a lethal gastrointestinal dose of vegetative *B. anthracis* (Sterne strain).

## 2. Results

### 2.1. Post-Exposure Administration of Anti-PA mAb Prevents Lethality in Mice with GI Anthrax

**Figure 1:**
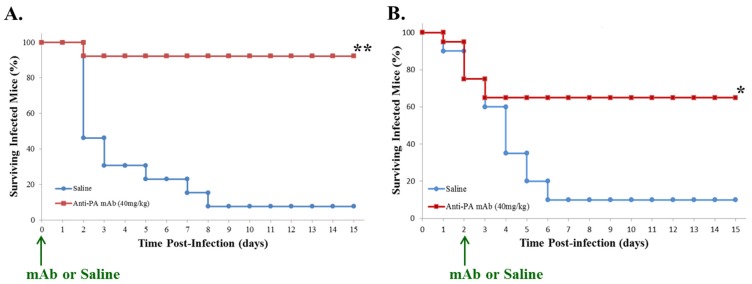
The effects of anti-anthrax protective antigen (PA) mAb on survival following gastrointestinal gavage with *B. anthracis*. (**A**) Mice received intravenous anti-anthrax PA mAb or saline (indicated by arrow) concurrently with a lethal dose of vegetative *B. anthracis* by oral gavage (~5 × 10^7^–5 × 10^8^ c.f.u.). Survival was monitored for fifteen days. (**B**) Mice were gavaged with lethal doses of vegetative *B. anthracis*, then administered anti-anthrax PA mAb or saline 48 h later (indicated by arrow). Survival was monitored for fifteen days. Statistical significance was calculated using the Mantel-Cox (log-rank) test (* *p* = 3.53 × 10^−3^; ** *p* = 1.19 × 10^−5^).

A/J mice were administered a lethal dose of vegetative *Bacillus anthracis* (Sterne) bacteria by oral gavage; intravenous treatment with a single dose of anti-PA mAb (40 mg/kg) or saline control was administered either concurrently or two days later. Treatment with anti-PA mAb at the time of anthrax exposure resulted in almost complete protection from fatality (Figure 1A), with >90% of mice (12/13) surviving the duration of the study (two weeks following anthrax challenge). In contrast, <10% of mice receiving the saline control (1/13) survived more than two weeks, with a great majority succumbing within seven days (Figure 1A). A second experiment revealed that delayed treatment with anti-PA mAb (48 h after gastrointestinal *Bacillus anthracis* gavage) also resulted in a survival advantage (13/20, 65%) (Figure 1B) compared to animals that received the saline control (2/20, 10%, over two weeks), although this effect was not as profound as that observed with immediate prophylactic therapy. All animals that died prior to the conclusion of the experiment were found to be blood culture positive, with culture results consistent with *Bacillus anthracis* infection. Some cultures also showed colony morphologies indicative of heterogeneous infections with enteric organisms, as we have previously reported to occur in mice treated with anthrax lethal toxin or infected with *Bacillus anthracis* [14,17]. In contrast, animals that survived the two-week post-infection period were found not to be bacteremic (data not shown).

### 2.2. Anti-PA mAb Treatment Blocks Intestinal Pathology Associated with GI Anthrax in Mice

**Figure 2:**
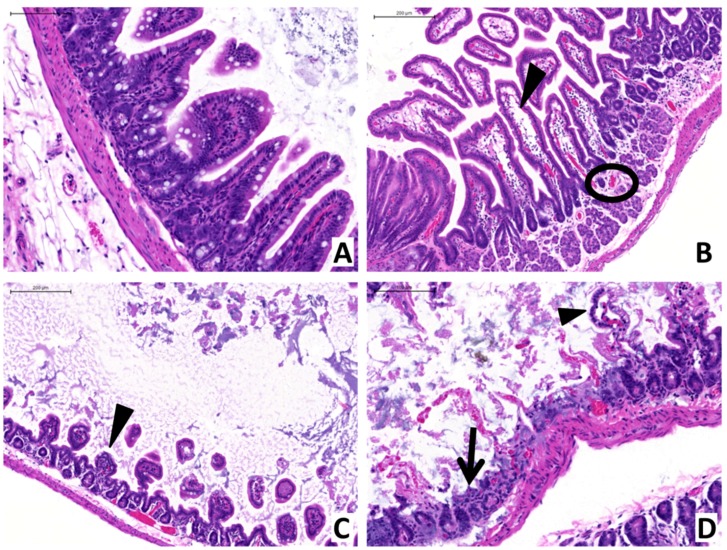
The effects of anti-anthrax PA monoclonal antibody on the histopathology present in small intestines of anthrax-gavaged mice. Small intestines were recovered from mice gavaged with lethal doses of *Bacillus anthracis* and treated with either anti-anthrax PA mAb (**A**) or saline (**B**,**C**,**D**), which were then fixed and sectioned for staining by H&E (representative images shown). (**A**) Intestines obtained from anti-anthrax PA mAb-treated animals exhibited a normal mucosal epithelium, with uniform microvillus architecture. (**B**) In contrast, intestinal sections obtained from control animals treated with saline showed segmental mucosal expansion due to edema (arrowhead), which was associated with congested blood vessels (circle). Lymphoid cells were occasionally observed in the villi and submucosa of these regions. (**C**) Microvilli atrophy was also observed within control animal intestines (arrowhead). (**D**) Furthermore, mucosal ulceration (arrow) and sloughing of the mucosal epithelium (arrowhead) were found in the intestines of control animals. Scale bars indicate 100 μm (**A**,**D**) or 200 μm (**B**,**C**).

**Figure 3:**
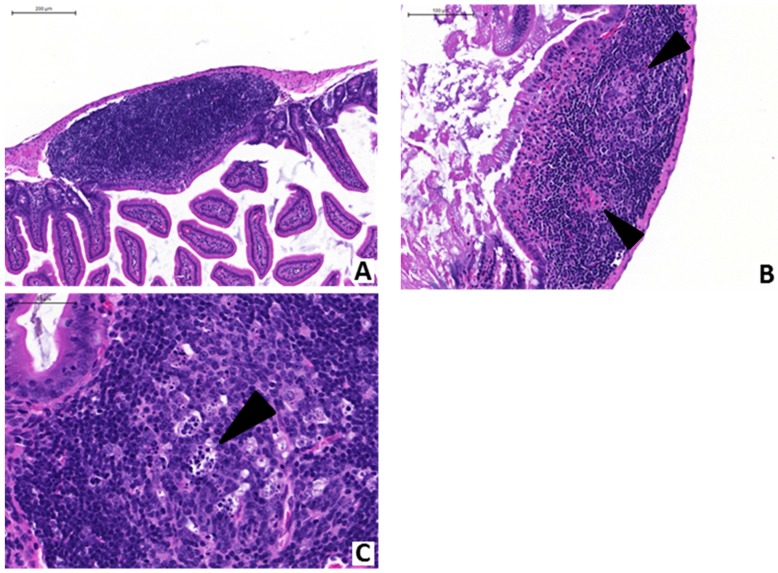
The effects of anti-anthrax PA monoclonal antibody treatment on the histopathology of enteric immune tissues from anthrax-gavaged mice. (**A**) Representative photomicrographs of enteric mucosal-associated lymphoid tissue (MALT) recovered from intestines of mice gavaged with anthrax and concurrently treated with anti-anthrax PA mAb showed no evidence of pathology (scale bar indicates 200 μm); (**B**) in contrast, infected control mice had MALT characterized by lymphoid depletion and prominent germinal centers (arrowheads; scale bar indicates 100 μm); (**C**) within the germinal centers of control mice, numerous macrophages with intracytoplasmic nuclear debris (tingible bodies, arrowhead) were observed (scale bar indicates 50 μm).

Anti-PA mAb (40 mg/kg) or saline control was administered intravenously to randomly-tagged pairs of A/J mice (one animal receiving mAb, the other receiving PBS) concurrent to gavage with vegetative *Bacillus anthracis*. Mice were subsequently examined at frequent intervals for characteristic signs of infection (reduced locomotor activity, subnormal grooming and hypothermia). Both mice of each pair were then euthanized upon development of morbidity in either animal. Dissection of euthanized animals was performed for recovery of intestines, which were then fixed and sectioned for hematoxylin and eosin (H&E) staining. Prior studies performed by our group have characterized the tissue damage and enteropathological consequences of anthrax LT and gastrointestinal anthrax infection on the murine intestinal tract [14,17,38]. Therefore, we sought to investigate whether anti-PA mAb mitigates tissue damage in the intestines of anthrax-infected mice. Examinations of enteric samples obtained from anthrax-gavaged mice that received anti-PA mAb revealed an absence of significant histopathological findings (Figure 2A). In contrast, enteric samples obtained from mice that were treated with saline (controls) revealed characteristic pathological changes (*i.e.*, villous edema, congestion, mucosal erosion and ulceration, mucosal atrophy and epithelial sloughing) (Figure 2B–D). Additionally, several infected control mice that were treated with saline exhibited lymphodepletion in their enteric mucosal-associated lymphoid tissues (MALT), in contrast to mice treated with anti-PA mAb, which displayed normal enteric MALT architectural structures (Figure 3). These histopathological findings are summarized in Table 1.

### 2.3. Anti-PA mAb Blocks the Penetration of B. anthracis into the Gut Epithelium

**Figure 4:**
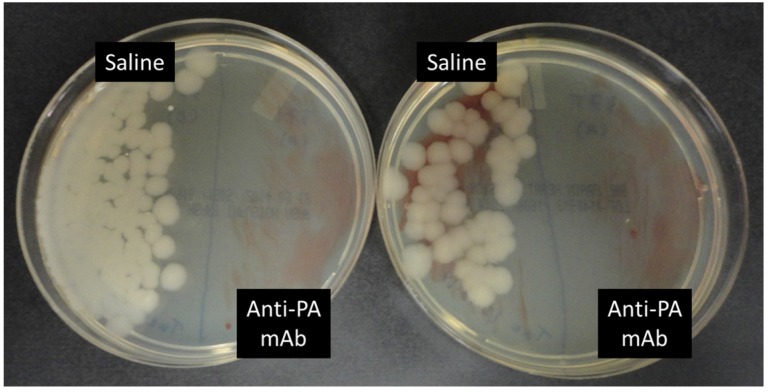
Representative images of blood culture results from paired experimental mice. Blood samples were obtained by sterile cardiac puncture from moribund mice gavaged with *B. anthracis* and treated with either saline or anti-anthrax PA in a paired treatment analysis (refer to the Experimental Section). Blood samples from moribund control mice that received saline treatment were smeared on the left half of each BHI agar plate as indicated; blood samples from mice that received anti-anthrax PA mAb-treatment were smeared on the right halves of the plates. Shown are the results from two representative moribund control (saline-treated) and two anti-PA-treated mice, collected two days after gavage. Blood from saline-treated controls showed rapidly growing colonies morphologically consistent with *Bacillus anthracis*, with evidence of occasional co-infection with commensal enteric bacteria, as previously described in Xie *et al.* (2013) and Sun *et al*. (2012) [14,17], whereas samples from anti-PA-treated mice showed no such growths.

**Figure 5:**
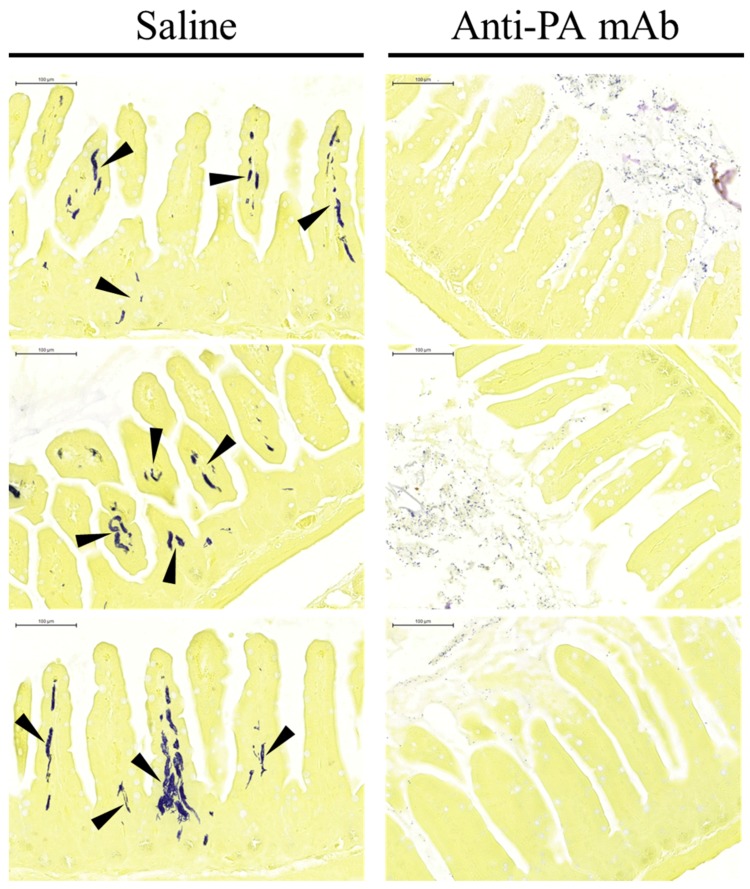
Photomicrographs of enteric tissue sections from experimental animals stained for bacteria. Small intestines recovered from anthrax-gavaged mice concomitantly treated with saline control (left column) or anti-anthrax PA mAb (right column) were fixed, then sectioned for staining by the Brown and Brenn method (representative images shown). Gram^+^ bacilli invading the tissue stain dark blue (arrowheads). Scale bars indicate 100 μm.

Blood cultures from *B. anthracis*-gavaged control mice revealed sepsis, while mice receiving anti-PA mAb therapy were found to have sterile blood cultures (Figure 4). Fixed intestinal specimens obtained from euthanized mice were also sectioned and stained by the Brown and Brenn (B&B) method for the detection and localization of bacteria within the recovered tissue. Bacterial invasion was noted in infected control mice, characterized by numerous clusters of colonizing anthrax bacteria within the villi and underlying submucosal layers, as previously described in Xie *et al.* (2013) [14] (Figure 5, Table 1). In some animals, bacteria had infiltrated the lymphatic ducts and blood vessels within the villi, providing access to routes of dissemination for migration to distant sites within the body. Strikingly, no instances of bacterial invasion were found in any of the samples obtained from anthrax-gavaged mice that were treated with the anti-PA mAb (Figure 5, Table 1), suggesting that the activity of anthrax toxin is required for the pathogen to overcome the gastrointestinal barrier, the initial step that subsequently progresses to lethal sepsis during gastrointestinal *B. anthracis* infection.

**Table 1:** The effects of anti-anthrax PA monoclonal antibody on intestinal pathology associated with gastrointestinal anthrax infection. Characterization of the intestinal pathology in mice from each treatment group (*n* = 5 per group) was based on the presence or absence of the indicated pathological findings in stained intestinal sections. Specimens were obtained from A/J mice gavaged with Bacillus anthracis (Sterne strain) and concomitantly treated with either anti-anthrax PA mAb or saline control. Animals were euthanized in pre-designated pairs (one mAb-treated and one saline-treated) upon development of morbidity by either animal in each pair. Values in the chart indicate the number of mice in each group (out of five) found to be exhibiting each of the specified pathologies (see Figure 2).

Pathological findings	Saline	Anti-anthrax mAb
Intra-mucosal invasion	5/5	0/5
Congestion	5/5	0/5
Villous edema	5/5	0/5
Lymphocytolysis	4/5	0/5
Mucosal erosion/ulceration	3/5	0/5
Enteric mucosal atrophy	2/5	0/5

## 3. Discussion

The fatal consequences of infection with *Bacillus anthracis* have been familiar to mankind since ancient times, with sporadic accounts of outbreaks afflicting humans and animals with coal-colored escharous skin lesions and darkened blood being recorded more than two thousand years ago [39,40]. The reemergence of weaponized anthrax from its nascent military development more than a century ago as a potential agent of biological warfare [41], to its recent exploitation as an instrument of bioterrorism, has presented the world with a renewed threat through its potential for mass civilian casualties [2]. Although the recently documented events of intentional anthrax exposure have thus far been limited to inhalation-directed attacks, the susceptibility of the general population via deliberate anthrax contamination of the food supply remains a vulnerability, as well [9,10]. Indeed, illnesses and fatalities due to consumption of contaminated meat continue to occur with some frequency in areas where anthrax bacilli are naturally endemic, such as Sub-Saharan Africa [42,43], the Middle East [44] and Southeast Asia [45]. In one instance, more than a hundred inhabitants of a village in western Uganda were sickened, and nine died within days of ingesting meat butchered from an anthrax-contaminated zebu (domestic cattle) [46]. Though not common, anthrax occasionally infects grazing ruminant livestock of North America, as well [46,47,48,49].

The possibility of an attack on the food supply by the purposeful introduction of anthrax has been a topic of speculation for some time, especially as past efforts to deliberately contaminate restaurant salad bars and grocery stores in Oregon with pathogenic bacteria, such as *Salmonella typhimurium*, have proven successful in spawning foodborne outbreaks [50,51]. The use of *Bacillus anthracis* in settings involving mass food preparation and consumption could prove devastating, as gastrointestinal infection with anthrax is often difficult to accurately diagnose in its early stages, and its symptoms can proceed too rapidly for the timely administration of therapy [10,52]. In this regard, the bactericidal activity of antibiotics has utility in the treatment of anthrax infection, and antibiotics are recommended for immediate administration to patients with systemic anthrax [52,53,54]. However, due to the inability of antimicrobial agents to inactivate the secreted exotoxins of *Bacillus anthracis*, numerous monoclonal antibodies specific for anthrax toxin components have been investigated as anthrax therapies [33], including the U.S.-licensed anti-PA mAb raxibacumab [33,34,36], which has shown its value in the setting of inhalational anthrax in animal models [32,34,36]. The present study demonstrates the substantial benefit of anti-PA mAb therapy in the setting of gastrointestinal anthrax exposure.

Our established experimental model utilizing the acapsular Sterne strain of *Bacillus anthracis* inoculated as vegetative organisms into complement-deficient A/J mice by oral gavage has allowed for the detailed analysis of gastrointestinal anthrax infection [14] and forms the basis for the current investigation. Our results have clearly demonstrated a significant survival advantage for mice receiving treatment with anti-PA mAb either concurrently with or up to forty-eight hours following gavage with *Bacillus anthracis*. Consistent with our previous results, nearly all mice receiving a lethal dose of vegetative *Bacillus anthracis* and saline alone (control group) became morbid and/or died in substantial numbers within two to six days. In contrast, nearly all mice that were concurrently administered anti-PA mAb therapy survived. It should be noted that a forty-eight hour delay in anti-PA treatment resulted in some deaths among the experimental animals during the two-day period immediately following bacterial gavage (similar to the rates seen in the control group). However, soon after the administration of anti-PA mAb therapy, fatal outcomes ceased, while the group receiving sham therapy continued to sustain a steady rate of fatalities. The finding that a significant number of animals could be protected from lethal gastrointestinal anthrax infection by anti-PA mAb treatment, even after a considerable delay, is an encouraging observation. This is suggestive of the potential benefit for anti-PA mAb therapy in a typical infection scenario where exposure might not be initially detected and treatment thereby delayed.

The successful treatment results achieved by the neutralization of *Bacillus anthracis* PA by anti-PA mAb in animal models of inhalational anthrax [33,34] confirm that many of the pathological processes associated with anthrax infection are dependent on the activity of the anthrax exotoxin, irrespective of the portal of infection. Moreover, this conclusion is in accordance with our previous observation that such enteropathologies as mucosal ulceration and erosion can be induced simply by the intravenous or intraperitoneal administration of anthrax LT, even in the absence of anthrax organisms [17]. Nevertheless, the site of action of anti-PA mAb activity has not been identified (e.g., intraluminal, epithelial, submucosal and/or in lymphoid tissues), indicating that further investigation in this area is warranted.

One of the most striking observations of the current investigation was the complete absence of detectable invasion by anthrax bacteria into the villi or submucosal layers of the small intestines of anti-PA mAb-treated animals (see Figure 5). This finding supports our previously-described model in which anthrax penetration through the gastrointestinal epithelial barrier is a key event in the pathogenesis of infection [17,38]. The subsequent invasion of bacteria into the villi and submucosa ultimately culminates in hematogenous distribution, leading to widespread dissemination of anthrax organisms and toxin to distant tissues in the body, resulting in the eventual failure of multiple organ systems [14]. The trans-epithelial invasion of anthrax has also been reported in a recent publication describing an alternative gastrointestinal infection model, which utilizes high numbers of *Bacillus anthracis* (Sterne) administered to A/J mice as a preparation of spores (up to 10^9^ per mouse), instead of vegetative organisms [55].

Although the processes through which anthrax toxins attenuate and dysregulate the immune responses to infection are becoming clear, the mechanistic roles for the toxin in promoting the initial stages of anthrax pathology remain enigmatic [18,23,25,26,56,57]. The activity of the anti-PA mAb in blocking anthrax invasion may at least be partially explained by the action of LT to induce a proliferative blockade of epithelial cells in the crypts of intestinal villi, leading to failure of villous structures [17]. In a tissue type such as the enteric epithelium, characterized by relatively rapid turnover, a disturbance in the usual sequence of cellular renewal at the apical layer would be predicted to interrupt barrier function, facilitating the invasion of pathogenic microorganisms. The inhibition of LT formation through PA neutralization by a specific mAb would therefore prevent this pathological process from occurring.

Another possible explanation for our observation of decreased enteric epithelial invasion by *Bacillus anthracis* following administration of anti-PA mAb might be drawn from our previous finding that anthrax LT stimulates the release of neutrophil elastase (NE) [38]. It has been proposed that NE may perform a pathogenic role in promoting a loss of integrity in the gastrointestinal epithelium, resulting in deterioration of its critical barrier function [58]. This, in turn, could facilitate the invasion of enteric microorganisms, including anthrax, into the villous submucosal layers, and eventually, into the circulatory system [17,59]. Moreover, anthrax LT has also been shown to interfere with major components of the cytoskeleton and microtubule system, adversely affecting cell-cell adhesion functions and barrier integrity in a model of lung epithelium [60]. These anthrax LT-induced cytoskeletal perturbations also hinder epithelial repair mechanisms, thus compounding the damage resulting from breaches in the epithelial layers [60]. Furthermore, in another system of physiological barrier maintenance, anthrax LT has also been shown to perturb endothelial integrity via downregulation of claudin-5, an integral protein of tight junctions [61,62], and disruption of VE-cadherin, an adhesion protein crucial to the formation of adherens junctions [62]. Future investigations focusing on these mechanisms are warranted to further elucidate the role of anti-PA mAb in protecting against lethal outcomes associated with gastrointestinal anthrax.

Gastrointestinal anthrax represents a persistent threat to livestock and humans who consume their meat. Moreover, the potential for a bioterrorism attack on the human or livestock food supply by deliberate contamination with anthrax remains a valid concern. The results described here demonstrate the clear benefit of anti-PA mAb administration for the prevention of the lethal consequences of gastrointestinal infection by *Bacillus anthracis* in an animal model of exposure. Additionally, they suggest a potential role in clinical settings where *Bacillus anthracis* exposures occur through ingestion of contaminated food under accidental or deliberate circumstances. In real-world exposure scenarios, the first-line therapy would be a regimen of sterilizing antibiotics; however, the data presented here support the potential benefit for a treatment plan that includes neutralizing anti-PA antibody, as well.

## 4. Experimental Section

### 4.1. Ethics Statement

All animal experiments were approved by the United States Food and Drug Administration Center for Biologics Evaluation and Research (CBER) Institutional Animal Care and Use Committee, which is endorsed by the Association for Assessment and Accreditation of Laboratory Animal Care International, and performed in accordance with the U.S. Public Health Service Policy on Humane Care and Use of Laboratory Animals (Assurance #A4295-01). Experimental animals were utilized in compliance with procedures detailed in Animal Protocol #WO2011-16 and were observed for any signs of illness or distress (hypothermia, rigor, diminished locomotor activity, respiratory difficulty, failure to groom) at least three times per day. Animals found to be exhibiting any of the aforementioned signs were euthanized by CO_2_ asphyxiation or cervical dislocation.

### 4.2. Gastrointestinal Challenge with Bacillus Anthracis Sterne Strain

Female A/J mice (9–12 weeks old, Jackson Laboratories, Bar Harbor, ME, USA) were housed in standard cages and acclimated for at least one week prior to commencement of the experiments. Rodent chow and water were provided *ad libitum* until initiation of a pre-gavage fasting period, during which chow was withheld for 16 h to permit evacuation of gastric contents [14]. Fasted mice were anesthetized by intraperitoneal injection of 60–70 mg/kg ketamine in combination with 12–14 mg/kg xylazine. Fifty microliters of 8.5% (*W/V*) NaHCO_3_ were then administered intragastrically for neutralization of peptic acid, immediately followed by gavage with the bacterial suspension containing ~5 × 10^7^–5 × 10^8^ c.f.u. vegetative *Bacillus anthracis* (Sterne strain 7702, seed stock kindly provided by Dr. Tod Merkel) in 150 µL brain heart infusion medium (BHI, BD 221813; Becton Dickinson and Company, Franklin Lakes, NJ, USA) [14]. All experiments using *Bacillus anthracis* (Sterne) were performed under Biosafety Level 2 conditions in accordance with approved protocols. Mice were subsequently returned to their cages for recovery, with resumption of chow and water *ad libitum*.

### 4.3. Therapeutic Agent

The anti-PA mAb that was utilized for all of the described *in vivo* studies was expired raxibacumab (GlaxoSmithKline, Brentford, U.K.), which had been cycled off of the Strategic National Stockpile after surpassing its expiration period. Biological activity was confirmed using an *in vitro* IL-2 production inhibition assay.

### 4.4. Murine Survival Study

Anti-PA mAb (diluted in isotonic saline [32]) or saline control was administered intravenously (40 mg/kg dosage, as previously approved for human use of U.S.-licensed raxibacumab) into the tail vein of mice, either concurrently or two days following gastrointestinal challenge with *Bacillus anthracis* (Sterne). Mice were examined at least three times per day for signs of morbidity. Animals that became morbid were euthanized by CO_2_ asphyxiation in euthanasia chambers or by cervical dislocation. Blood (~10–20 µL) was immediately drawn by aseptic cardiac puncture using 26 G × 5/8” needles (BD 309597, Becton Dickinson and Company), then diluted in 100 µL of sterile PBS for culture on BHI agar plates (BD 221569, Becton Dickinson and Company) to assess the presence of bacteremia. Animals that survived up to 15 days post-gastrointestinal challenge were similarly euthanized for analysis.

### 4.5. Paired Treatment Analysis

In advance of the experiment, A/J mice were randomly tagged, and pre-assigned inter-group treatment pairs were established. Subsequently, one mouse from each pair was administered intravenous anti-PA mAb concurrently with gastrointestinal challenge with *Bacillus anthracis* (Sterne), while the designated corresponding mouse in each pair was administered intravenous saline (control) concurrently with gastrointestinal *Bacillus anthracis* (Sterne) challenge. All mice were observed as previously described (see above), and any animals found to be moribund were euthanized and dissected to obtain cardiac blood samples (for culture) and small intestines, which were subsequently fixed in 10% neutral buffered formalin. Additionally, the pre-designated inter-group partner in each pair was simultaneously euthanized and similarly assessed for control purposes.

### 4.6. Pathology Assessment

The fixed small intestines (see above) were sectioned and stained by the hematoxylin and eosin (H&E), or Brown and Brenn (B&B) methods (Histoserv Inc., Germantown, MD, USA) as previously described [14,17,38]. Stained slides were scanned using a Pannoramic MIDI system (3DHistech Kft., Budapest, Hungary). The assessment of enteropathologic injury and invasion by *Bacillus anthracis* organisms was performed in a blinded manner by a boarded veterinary pathologist (D. Rotstein).

### 4.7. Statistical Methods

Statistical analysis of experimental data was performed using the Microsoft Excel (Microsoft Co., Redmond, WA, USA) and GraphPad Prism 5 (GraphPad Software, Inc., La Jolla, CA, USA) software packages. For survival experiments, the log-rank (Mantel-Cox) test was applied.

## 5. Conclusions

This study demonstrated that the intravenous administration of a monoclonal antibody that neutralizes the activity of the protective antigen component of anthrax toxin is an effective stand-alone post-exposure therapeutic in a mouse model of intestinal *Bacillus anthracis* infection. Passive immunity via this therapeutic imparts protection against the enteropathological effects of anthrax toxin in the gastrointestinal system and blocks invasion of *Bacillus anthracis* into the enteric submucosa, thus preventing subsequent lymphohematogenous dissemination. These results suggest that anti-PA mAb therapy could contribute to a successful strategy for the prevention or treatment of gastrointestinal anthrax infection, even if therapy is delayed following exposure.
